# Affinity Capture Elution (ACE) ELISA Method Development and Validation for Novel RPH-104 Drug Immunogenicity Evaluation

**DOI:** 10.3390/biomedicines10112750

**Published:** 2022-10-29

**Authors:** Maria A. Kolganova, Elizaveta V. Melnik, Elizaveta N. Fisher, Valery V. Smirnov, Alexander M. Vlasov, Vladimir I. Gegechkori, Nikolay A. Shulga, Igor E. Shokhin, Galina V. Ramenskaya

**Affiliations:** 1 LLC ‘Center of Pharmaceutical Analytics’, 117246 Moscow, Russia; 2 A.P. Nelyubin Institute of Pharmacy, I.M. Sechenov First Moscow State Medical University (Sechenov University), 119991 Moscow, Russia

**Keywords:** interleukin-1, RPH-104, ADA, ELISA, ACE, immunogenicity, bioanalytical method, validation

## Abstract

As the number of therapeutic protein products is growing rapidly, there is a strong need for the development of bioanalytical methods that are easy to perform, specific, sensitive, robust, and affordable. Methods for immunogenicity evaluation of therapeutic proteins take an important place in this field of bioanalytics. The aim of the study was to develop a method for immunogenicity testing of the novel RPH-104 drug using the Affinity Capture Elution (ACE) ELISA technique. RPH-104 is a promising Interleukin-1 (IL-1) inhibitor that is currently undergoing a series of clinical studies, including those on socially significant and orphan diseases. The developed method was validated for assay cut-point, sensitivity, selectivity, drug tolerance, hook effect, specificity, precision, and stability. Method sensitivity was established at 114.9 ng/mL, while low and high positive controls were equal to anti-RPH-104 antibody concentrations of 155 ng/mL and 2500 ng/mL, respectively. Method specificity was confirmed in the presence of the interfering compounds, namely IL-1α, IL-1β, and IL1-Ra. The developed and validated ELISA method was successfully applied to subject samples.

## 1. Introduction

The family of Interleukin-1 (IL-1) currently comprises 11 pro- and anti-inflammatory cytokines [1,2,3]. Among them, two potent pro-inflammatory mediators, IL-1α and IL-1β, have been, in particular, thoroughly studied in the recent decade, emphasizing their significant role in the inflammatory process [4]. All cells and organs are involved in IL-1 signaling pathways that predetermine a key role of IL-1 in autoinflammatory, autoimmune, infectious and degenerative diseases [3].

Although IL-1α and IL-1β are characterized by similar biological properties, there are several important distinctions that affect their function role in living organisms. Thus, IL-1α is constantly present as a precursor protein in epithelial layers of organs such as lungs, liver, gastrointestinal tract, and kidneys, as well as in endothelial cells and astrocytes. Moreover, IL-1α is located in a membrane form on activated monocytes. IL-1α precursor is released into the extracellular space upon necrotic cell death, while, being bioactive, it immediately initiates the inflammatory process. On the contrary, IL-1β is produced in an inactive precursor form by hematopoietic cells such as monocytes, macrophages, and dendritic cells in response to immune cells’ exposure to alarmins. Thereafter, IL-1β precursor is activated upon cleavage by caspase-1, a cysteine protease. Both IL-1α and IL-1β bind to the same IL-1 receptor type 1 from the IL-1 receptor family (commonly referred to as IL-1R1). The IL-1 receptor accessory protein (RAcP, IL-1R3) is a coreceptor necessary for IL-1R signaling via receptor complex formation with IL-1R1. In contrast to IL-1R1, IL-1 receptor type 2 (IL-1R2) inhibits IL-1 signaling by preventing IL-1 binding to IL-1R1 and preventing IL-1 mediated effects [1,2,3,4].

Consequently, the increase in IL-1 expression can be associated with diverse inflammatory signals as a physiological norm. However, IL-1 upregulation is known to be crucial for the development of a wide range of auto-inflammatory and autoimmune diseases [3,5,6,7]. IL-1α has been identified as a driver for cutaneous, neural, colon inflammation, colon cancer and cardiovascular diseases, apart from its role in myocardial infarction and brain-injury-induced ischemia [5]. In its turn, an increased release of active IL-1β plays a key role in a number of common diseases such as rheumatoid arthritis, type 2 diabetes, smoldering multiple myeloma, postmyocardial infarction heart failure, osteoarthritis, etc. [7] Such diseases as apatite-associated arthropathy, Behçet disease, gout, Still disease as well as genetically driven autoinflammatory disorders such as familial Mediterranean fever (FMF), familial cold-induced autoinflammatory syndrome (FCAS), cryopyrin-associated periodic syndromes (CAPS) are strongly associated with IL-1 production and/or functional activity disorders [8].

The therapeutical potential of addressing IL-1 has been realized with IL-1 inhibitors, namely anakinra, rilonacept, and canakinumab. Rilonacept represents a recombinant protein that incorporates the IL-1R1 and the IL-1 RAcP domains in one molecule, neutralizing both IL-1α and IL-1β [9]. Anakinra is analogous to a naturally occurring IL-1 receptor antagonist (IL-1Ra) that acts by blocking the interaction of IL-1 with IL-1R1 [10]. Unlike previous IL-1 inhibitors, canakinumab is a high-affinity human monoclonal anti-IL-1β antibody that specifically binds to IL-1β and inhibits IL-1β mediated effects [11].

A recent addition to the IL-1 antagonist range is RPH-104, a heterodimer fusion protein comprising two receptor components, binding IL-1α and IL-1β: extracellular IL-1R1 domain and a part of IL-1 RAcP [12]. It is a promising biotherapeutic agent due to its small size and high stability compared to other IL-1 inhibitors, besides having a strong affinity to both IL-1α and IL-1β [13]. Nowadays, a number of clinical studies on RPH-104, including socially significant and orphan diseases, are carried out.

While opening up new treatment possibilities, biopharmaceuticals often encounter a number of limitations. One of the issues common for therapeutic protein products is drug immunogenicity manifestation that is characterized by anti-drug antibodies (ADA) formation [14,15]. ADA can significantly reduce drug efficacy by altering its pharmacokinetics along with pharmacodynamics up to triggering serious adverse reactions.

Monitoring therapeutic protein immunogenicity is crucial for evaluating drug safety and efficacy. For this purpose, a number of analytical techniques based on enzyme-linked immunosorbent assay (ELISA) are currently used [16]. Among them, one of the common approaches is affinity capture elution (ACE) ELISA [17,18]. It is a drug-tolerant assay that is able to detect ADA in the presence of a large amount of circulating drug by performing two acid dissociation steps. One step is required for the disruption of an ADA-drug complex with a subsequent affinity capturing of ADA on a solid-phase drug, while through the second step, ADA are released, transferred onto a second carrier, and detected by a biotinylated drug. Despite having an improved drug tolerance, the ELISA method should be applied carefully because the two-step dissociation process can cause assay variability.

The aim of this study was to develop and validate a bioanalytical method for the detection of ADAs for RPH-104 in blood serum samples by ELISA. The developed and validated method was successfully applied to real samples obtained from a clinical study of RPH-104 efficacy and safety in patients with idiopathic recurrent pericarditis.

## 2. Materials and Methods

### 2.1. Reagents

The studied therapeutic protein RPH-104 was supplied by R-Pharm JSC (Moscow, Russia) in a solution form with a measured concentration of 38.6 mg/mL. Affinity-purified rabbit anti-RPH-104 monoclonal antibodies (used as a positive surrogate control for this assay) were obtained from Covance Research Products (Harrogate, UK). Horseradish peroxidase (HRP) conjugated streptavidin and 3, 3′, 5, 5′ tetramethylbenzidine (TMB) substrate were purchased from Imtek (Moscow, Russia). Bovine serum albumin (BSA) heat shock fraction, pH = 7, was obtained from Sigma Aldrich (St. Louis, MO, USA). Human recombinant IL-1α and human recombinant IL-1β were purchased from BioLegend (San Diego, CA, USA), human recombinant IL-1Ra was obtained from R&D Systems (Minneapolis, MN, USA). Human recombinant IL-1α, IL-1β, and IL-1Ra were used only for validation purposes to assess method specificity.

Tween-20 pure pharma grade was purchased from PanReac (Castellar del Vallès (Barcelona), Spain), glycine was purchased from Dia-M (Moscow, Russia), and tris(hydroxymethyl)aminomethane was obtained from Sigma Aldrich (St. Louis, MO, USA). Sodium carbonate, sodium bicarbonate, potassium chloride, sodium chloride, potassium dihydrogen phosphate, disodium hydrogen phosphate, and sulfuric acid pure analytical grade were purchased from the local distributor (Moscow, Russia). Type 1 water was obtained in-house using the water treatment system Aqualab AL-1 Plus by Mediana-Filter (Moscow, Russia).

The wash buffer was composed of 0.1% Tween-20 in 1X Phosphate-Buffered Saline (PBS, prepared in-house). The assay buffer was freshly prepared by dissolving BSA and Tween-20 in 100 mL 1× PBS (0.05% Tween-20 with 1% BSA in PBS).

### 2.2. Biotin-RPH-104 Conjugate

The biotin-labeled drug was obtained in-house. The labeling of RPH-104 was performed using Antibody Biotinylation Kit (Sileks, Moscow, Russia). The prepared reagent pre-mixture from the kit was added to 1 mL of RPH-104 solution and incubated overnight. The prepared biotinylated drug was then freshly diluted at 1:200 in assay buffer for further use as a primary detection reagent.

### 2.3. Positive and Negative Controls

Positive controls (PC) at different ADA levels were generated by adding anti-RPH-104 antibodies to intact pooled human sera obtained from treatment-naïve subjects. Briefly, the anti-RPH-104 antibody sera stock sample (10,000 ng/mL) was firstly prepared by diluting the anti-RPH-104 antibody stock solution (1.09 mg/mL) with intact pooled human sera. Then, high and low PCs (HPC and LPC) with a concentration of 2500 ng/mL and 155 ng/mL, respectively, were obtained by diluting the sera stock solution with intact sera. After that, HPC and LPC samples were incubated for 1 h at 37 °C, 500 rpm (modeling anti-RPH-104 antibodies distribution in patient blood) and stored at a temperature ≤ −20 °C for at least 12 h.

Intact pooled human sera were obtained in-house from treatment-naïve subjects and used as a negative control sample for this analytical method (NC).

### 2.4. ACE ELISA Assay

Briefly, during the assay, 96-well clear flat bottom polystyrene high bind microplates (Corning Inc, Corning, NY, USA) were coated overnight at 2–8 °C with RPH-104 solution (50 µg/mL). All samples (test samples, PCs, NCs) were thawed at room temperature (RT) for at least 30 min. Then, they were acidified with glycine buffer for 25 min at 37 °C and 500 rpm. All plate wells were washed 4 times with 350 µL of wash buffer using automated plate washer Aquamarine (BioSan SIA, Riga, Latvia). After washing, 50 µL of Tris-HCl buffer (for screening assay) or Tris-HCl buffer with RPH-104 (1 mg/mL, for confirmatory assay) were added to coated wells, and then, 100 µL of acid-treated samples were transferred into microplate wells in duplicates. Microplates were further incubated for 1 h at 37 °C and 500 rpm. After 4 washing cycles, as described above, the elution of bound anti-RPH-104 antibodies (ADAs) was performed by the addition of glycine buffer (pH = 2.30). The microplate was incubated for 25 min at 37 °C and 500 rpm. After incubation, fresh polystyrene microplates were loaded with 50 µL Tris-HCl buffer, and eluted samples (100 µL) were transferred into these plates. Microplates were incubated as described above for binding of the eluted anti-RPH-104 antibodies to the wells. After washing, plates were blocked by 250 µL/well of blocking solution (1% BSA) for 45 min at 37 °C and 500 rpm. The plates were then again washed 4 times, and primary detection reagent (biotinylated RPH-104) was added to all wells, followed by incubation for 45 min at 37 °C, 500 rpm. After washing, 100 µL of secondary detection reagent (HRP conjugated streptavidin) was added and incubated for 45 min at 37 °C and 500 rpm. Plates were washed the same as above, followed by 100 µL/well of TMB substrate solution and a 15-min incubation at RT (light protected). After 15 min, the reaction was stopped with 100 µL of 2N sulfuric acid, and the plate absorbance was read at 450 nm/630 nm using the Microplate Reader Stat Fax 3200 (Awareness Technology, (Palm City, FL, USA).

### 2.5. Method Validation

The developed method was validated according to the FDA guidance for immunogenicity testing of therapeutic protein products [19] for assay cut-point, sensitivity, selectivity, drug tolerance, hook effect, specificity, precision, and stability.

#### 2.5.1. Assay Cut-Point

Cut-point was determined for both screening and confirmatory assay formats in compliance with FDA recommendations and “classic” approaches widely described in the literature [20]. In order to estimate the cut-point values, 50 individual samples of blank human serum, including the samples from treatment-naïve subjects, were analyzed according to the developed ELISA method simultaneously in screening and confirmatory assay formats. The cut-point runs were performed by two analysts for five days. Apart from samples from treatment-naïve subjects, every run included PC (in concentrations of 2000 and 500 ng/mL) as well as NC samples. As a result of the cut-point determination normalization factor (NF) for screening, floating cut-point and fixed confirmatory cut-point were calculated and finalized for further assay.

#### 2.5.2. Sensitivity and Hook Effect

Assay sensitivity was determined together with HPC and LPC concentration establishment. The analytical assessment was performed during two runs by two analysts for a series of PCs with a range of anti-RPH-104 antibody concentrations as follows: standards 10-1 with concentrations of 50, 75, 100, 250, 500, 1000, 2500, 5000, 10,000, and 25,000 ng/mL. Each run comprised 3 dilution series (standards 10-1), 3 PC sets (in concentrations of 2000 and 500 ng/mL) and NCs. The hook effect was also evaluated for the developed method due to the inclusion of samples with high anti-RPH-104 antibody concentrations (up to 25,000 ng/mL, standard 1) in sensitivity determination runs.

#### 2.5.3. Selectivity

Assay selectivity was established through two analytical runs, each of them containing 5 individual samples of blank human sera with (HPC, LPC) and without (NC) addition of anti-RPH-104 antibodies. The selectivity samples were analyzed in both screening and confirmatory assay formats.

#### 2.5.4. Drug Tolerance

Drug tolerance was assessed in order to evaluate the method’s ability to obtain sustainable results and detect antibodies in the presence of therapeutic drug protein. For this purpose, 25 samples containing anti-RPH-104 antibodies and various concentrations of the drug were analyzed in screening assay format. Detailed information on the sample content is presented in Table 1. Anti-RPH-104 antibody and drug-containing samples were prepared in a two-time concentration using the biological matrix (intact pooled human sera from treatment-naïve subjects). Afterward, they were mixed in a ratio of 1:1 to obtain samples for drug tolerance evaluation.

#### 2.5.5. Specificity

Method specificity was assessed in the presence of the interfering compounds, namely IL-1α, IL-1β, and IL1-Ra. Furthermore, specificity was evaluated in the presence of the studied RPH-104 drug in a concentration not higher than the confirmed method drug tolerance level (75 µg/mL). The following samples were prepared using blank pooled human sera: samples of the interfering compounds, PCs and RPH-104 samples in a three times concentration (all in biological matrix). Afterward, the samples were mixed in a ratio of 1:1:1 in order to obtain samples with the required concentrations of different compounds for specificity establishment. The samples were prepared according to Table 2.

#### 2.5.6. Precision

Method precision was determined for both screening and confirmatory assay formats. HPC and LPC samples were analyzed during 6 runs by 2 analysts in 3 days. Each run involved at least 3 sets of quality controls (HPC, LPC, and NC). The first run for the first analyst comprised 6 sets of quality controls for intra-day precision assessment. Data obtained during all 6 runs were used for inter-day precision determination.

Both intra-day and inter-day precisions were calculated as a coefficient of variation (CV, %) for the first run and all 6 runs, respectively. Plate-specific cut point (PSCP) value was calculated for each cycle using values of any 4 NC samples (only them) among all samples analyzed in that cycle. The screening protocol precision was evaluated by mean OD values of HPC and LPC. The confirmatory protocol precision was assessed using calculated values of % inhibition.

#### 2.5.7. Stability

Method validation also involved anti-RPH-104 antibody stability examination under different handling and storage conditions. Bench-top stability was evaluated in PCs after storage for 15 h at room temperature (18–25 °C). Freeze-thaw stability was assessed after three freeze-thaw cycles; each freezing was not less than 12 h. Long-term stability studies were performed after low-temperature freezing of PCs at temperatures from −65 °C to −80 °C. Bench-top and freeze-thaw stability cycles encompassed 2 sets of freshly prepared PCs for PSCP determination and system suitability control. Moreover, these runs included 3 sets of PCs for each type of stability studies to evaluate anti-RPH-104 antibody stability.

### 2.6. Data Analysis

Statistical data analysis was applied to OD values obtained for individual blank serum samples during cut-point assessment. The data were processed using SPSS statistics software and MS Excel.

### 2.7. Application to Subject Samples

The developed and validated ELISA method was applied to idiopathic recurrent pericarditis patient samples of human blood serum. There were 22 patients enrolled in the study. The total number of samples was 201. Samples of the examined blood serum were stored in a freezer at a temperature from −65 °C to −80 °C.

The analytical procedure involved a screening assay with the subsequent confirmatory analysis and antibody titer determination. PSCP value was established for each run (plate).

All study samples with a mean OD greater than or equal to the PSCP were considered “screening positives” during the screening assay and were further analyzed in a confirmatory assay format. During the confirmatory analysis, the samples were analyzed simultaneously according to the protocol of screening and confirmatory analysis. Next, the % inhibition was calculated using the average values of the sample OD with and without the addition of RPH-104. Samples with % inhibition greater than 37.23% (confirmatory cut-point) were considered “confirmed positives” by the confirmatory assay. All samples were analyzed in duplicates.

## 3. Results

### 3.1. Method Development

During method development, we checked the background noise and signal-to-noise ratio for different concentrations of critical reagents. Several concentrations and dilutions were tested for coating reagents, primary and secondary detection reagents. The method showed optimal results at 50 µg/mL of RPH-104 for plate coating, 1:200 and 1:2500 dilutions for primary and secondary detection reagents, respectively.

To enhance method drug tolerance, we compared the two most commonly used acidifying agents—glycine buffer and 300 mM acetic acid solution, choosing glycine buffer (pH = 2.30), as it showed better results for negative control samples. As the acidification step can be crucial for method results variability, we also checked the optimal time and conditions of the sample acidification and ADAs elution steps, varying the acid incubation time from 5 to 45 min.

As the final step of the method development, the MRD (minimal required dilution) was finalized at 1:15 in glycine buffer for each sample (PCs, NCs, or test samples), taking into account further dilution of the sample at the neutralization step, when the Tris-HCl buffer is added to the plate wells. Pre-validation runs for selectivity and precision confirmed the developed conditions could be used for further method validation, so the analytical method ELISA was finalized and prepared to validation.

### 3.2. Method Validation

#### 3.2.1. Assay Cut-Point

To determine the assay cut-point for the screening assay, statistical processing of the obtained results was carried out. Firstly, all the data obtained (mean OD values of Set 1-Set 3, 50 samples each, for 2 analysts—total N = 300) were checked for normal data distribution using the Shapiro–Wilk test. The data were not normally distributed, so it was log-transformed and subjected to the procedure of statistical outlier elimination using Tukey criteria: all values not included in the range [Q1 − (1.5 ∗ IQR)]; [Q3 + (1.5 ∗ IQR)], where Q1 and Q3 are the first and third quartiles, respectively, and IQR is the interquartile range, were excluded from statistical analysis. Following outlier exclusion, a re-test for normality was undertaken by the Shapiro–Wilk test. The data were distributed normally, so the calculation of the fixed (validation) cut-point for the screening stage was conducted according to the formula (1):

CP.V = Mean_1_ + 1.645 ∗ SD_1_ (1)

where CP.V is the value of the fixed (validation) cut-point for the screening stage; mean_1_ is the mean OD value of all intact human blood serum samples obtained at the screening procedure after outlier exclusion; SD_1_ is the standard deviation of all intact human serum samples obtained at the screening procedure after outlier exclusion.

The value of the fixed cut-point for the screening stage was finalized at 0.036. Afterwards, the homogeneity of variances (using Levene’s test) and the comparison of means (using one-way analysis of variance (ANOVA) was assessed between runs and between analysts. Homogeneity of variances was observed both between analysts and between runs (*p* > 0.05), as well as statistical homogeneity of means was revealed both between analysts and between runs (*p* > 0.05). Based on the data obtained, a floating screening cut-point, as less strict and more common type of screening cut-point was chosen for further use in the validation and analytical assay. To confirm a screening floating cut-point application possibility, the Pearson correlation for the NC samples included in each cycle and the mean values for individual intact human serum samples for each cycle were calculated. A correlation value of more than 0.7 (0.768) confirmed the possibility of using a screening floating cut-point and the applicability of chosen pooled blank human sera as a negative control for this method. NF value was also calculated for the use of the floating cut-point. NF, in turn, was further utilized to calculate PSCP for each run. The calculated NF value was 0.008. During the subsequent validation and analytical assays, PSCPs were calculated as the average value of the NC samples OD plus NF.

Calculated% inhibition values (N = 300) were used to determine the cut-point for the confirmatory assay. The obtained results were subjected to statistical processing. First, the data were tested for normality using the Shapiro–Wilk test, and the data distribution was not normal. Then, the values were log-transformed and statistical outliers were eliminated—values not included in the range [Q1 − (1.5 ∗ IQR)]; [Q3 + (1.5 ∗ IQR)], where Q1 and Q3 are the first and third quartiles, respectively, and IQR is the interquartile range, were excluded from statistical analysis. After outlier exclusion, a re-test for normality was carried out using the Shapiro–Wilk test. The data were distributed normally. The fixed confirmatory cut-point was calculated using the formula (2):

Confirmatory CP = Mean_2_ + 2.33 ∗ SD_2_ (2)

where confirmation CP is the value of the fixed exclusion limit for confirmatory analysis; mean_2_ is the average value of the calculated values of % signal inhibition; SD_2_ is the standard deviation of the calculated values of % signal inhibition. The value of the fixed confirmatory cut-point was finalized at 37.23%.

#### 3.2.2. Sensitivity

Mean OD values obtained through method sensitivity assessment, as well as LPC and HPC concentration determination, are presented in Table 3.

For each of the two plates, the PSCP value was calculated. For plate 1, it was 0.035; for plate 2, the PSCP value was 0.034. To determine the assay sensitivity and the LPC concentration, the nominal concentrations of samples characterized by a higher average OD than the value of the corresponding PSCP were used. The concentration values (ng/mL) were log-transformed, and then the sensitivity of the method was calculated using the following formula (3):

Sensitivity, ng/mL = average concentration (log) + (t_0.05,df_ × S.D.) (3)

where t_0.05,df_ is a one-tailed Student’s t-test corresponding to a 5% error probability (OD below PSCP).

Conversion of the obtained value from the log form into the original scale enabled to obtain a numerical value of the method sensitivity. Method sensitivity calculations are shown in Table 4.

The LPC concentration was also determined using the concentration values of the samples with a mean OD value higher than PSCP. The concentration values (ng/mL) were converted to log form, and then the LPC concentration was calculated using formula (4):

LPC, ng/mL = average concentration (log) + (t_0.01,df_ × S.D.) (4)

where t_0.01,df_ is one-tailed Student’s t-test corresponding to 1% error probability (OD below PSCP).

The conversion of the obtained value from the log form to the original enabled to obtain the numerical value of the LPC concentration.

The calculated method sensitivity was 114.9 ng/mL. The theoretical concentration of the LPC sample was 154.9 ng/mL. As a matter of convenience and accuracy during control preparation, an anti-RPH-104 antibody concentration of 155 ng/mL was chosen as the practical concentration of LPC. The HPC concentration was selected as the upper point of the calibration curve in the linear range and was equal to the anti-RPH-104 antibody concentration of 2500 ng/mL.

#### 3.2.3. Hook effect

The calculated signal-to-noise (S/N) ratio in relation to the corresponding PSCP values for all standards is presented in Table 5. The results obtained demonstrate the absence of hook effects since no reduction in standard OD was observed with the increase in anti-RPH-104 antibody concentration.

#### 3.2.4. Selectivity

For each plate, the PSCP value was determined, which was equal to 0.034 for the first plate and 0.042 for the second plate. Selectivity results are provided in Table 6. Method selectivity was confirmed since 90% of individual intact serum samples had OD values lower than PSCP. At the same time, 100% of PCs (HPC, LPC) were confirmed positive according to screening and confirmatory assay results.

#### 3.2.5. Drug Tolerance

Mean OD values as well as RPH-104 analysis calculations are available in Table 7 (PSCP = 0.049). Method drug tolerance is quantified as a maximum drug concentration when signal stays above PSCP at various anti-RPH-104 antibody concentration levels. Drug tolerance for the developed method was an RPH-104 concentration of 200 µg/mL at an anti-RPH-104 antibody level of 250 ng/mL.

#### 3.2.6. Specificity

The PSCP value for the specificity assessment run was 0.052. Specificity determination results are presented in Table 8. Analysis results demonstrated that the mean OD values of the PCs were higher than the calculated PSCP value. The method specificity was confirmed, meaning that anti-RPH-104 antibodies can be determined in the presence of IL-1α, IL-1b, IL-1Ra, and RPH-104 drugs.

#### 3.2.7. Precision

Intra- and inter-day precision results are presented in Table 9 and Table 10, respectively. Calculated CV values did not exceed 20%, being within the acceptable limits according to [19] requirements.

#### 3.2.8. Stability

PSCP value for the bench-top (BTS) and freeze-thaw (F/T) stability run was equal to 0.039. CV (%) was calculated using mean sample OD values among three sample sets for each type of stability assay. The samples were considered stable through bench-top, and freeze-thaw stability evaluation as CV values did not exceed 20%. Stability examination results are presented in Table 11.

Long-term stability was assessed with intermediate results at the points of 1, 3, and 6 months and the final result in 14,5 months. Intermediate long-term stability (30-, 60-, and 209-day storage in a freezer at the temperature between −65 °C and −80 °C) results are presented in Table 12. The final evaluation of long-term stability was carried out after 414 days of storage in a freezer at the temperature specified above. Final long-term stability results are also enclosed in Table 12. PSCP values were 0.049, 0.044, 0.031, and 0.042 for 1-, 3-, 6-, and 14.5-month stability, respectively. The samples were considered stable for 30-, 60-, 209-, and 414-day storage since the CV values did not exceed 20%.

### 3.3. Application to Subject Samples

The screening and confirmatory cut-points established during method validation were used in patient blood serum analysis to differentiate study samples into screening and confirmed positives based on the comparison of the OD and % inhibition results acquired for the patient samples with the appropriate assay cut-point.

Based on the screening results, 10 samples out of 201 (samples from 5 patients out of 22 patients) were considered screening positive for the presence of anti-RPH-104 antibodies. Samples found to be screening positive were analyzed in a confirmatory test. Based on the confirmatory analysis results, six samples of the patient blood serum (2.98%) containing antibodies to RPH-104 were detected (samples from 3 patients out of 22 patients).

Confirmed positive samples were further analyzed at various dilutions to determine the titer of specific anti-RPH-104 antibodies there.

Thus, based on the analysis results, six samples of patient blood serum (3 patients out of 22, 13.6%) containing anti-RPH-104 antibodies were identified.

The information about the method application and sample analysis is finalized in Table 13.

## 4. Discussion

The developed method was fully validated in accordance with the FDA guidance for immunogenicity testing of therapeutic protein products [19] and successfully applied to anti-RPH-104 antibody measurement in RPH-104 treated patients. The apparent advantage of the described method is its simplicity of execution, allowing to monitor ADA formation in treated patients quickly and cost-effectively. Moreover, the assay is drug-tolerant, which enables the detection of anti-RPH-104 antibodies in the presence of large amounts of the drug (RPH-104 concentration of 200 µg/mL at an anti-RPH-104 antibody level of 250 ng/mL).

Since the developed and validated method involves an acid dissociation step, assay limitations such as high background noise, sensitivity loss, and high false-positive result level could be anticipated [16]. However, the method sensitivity was highly satisfactory (114.9 ng/mL), and the screening assay detected only 4 false positive results out of 201 samples (1.99%) that were cut off during confirmatory test.

Therefore, the validated method may be of considerable importance as a part of decision support system for clinical efficacy evaluation of RPH-104 in particular (including orphan disease treatment) and protein drugs in general.

## Figures and Tables

**Table 1:** Sample composition for drug tolerance examination.

Sample No.	Initial Concentration of Anti-RPH-104 Antibodies, ng/mL	Initial Concentration of RPH-104, µg/mL	Final Concentration of Anti-RPH-104 Antibodies, ng/mL	Final Concentration of RPH-104, µg/mL
1	500	0	250	0
2	500	200	250	100
3	500	400	250	200
4	500	800	250	400
5	500	1000	250	500
6	1000	0	500	0
7	1000	200	500	100
8	1000	400	500	200
9	1000	800	500	400
10	1000	1000	500	500
11	2000	0	1000	0
12	2000	200	1000	100
13	2000	400	1000	200
14	2000	800	1000	400
15	2000	1000	1000	500
16	5000	0	2500	0
17	5000	200	2500	100
18	5000	400	2500	200
19	5000	800	2500	400
20	5000	1000	2500	500
21	10,000	0	5000	0
22	10,000	200	5000	100
23	10,000	400	5000	200
24	10,000	800	5000	400
25	10,000	1000	5000	500

**Table 2:** Preparation of samples for specificity evaluation.

Concentration of Interfering Compounds, pg/mL		RPH-104, µg/mL	PC Concentration, ng/mL		
			0	155	2500
IL1-α	0.00	0.00	Blank	LPC	HPC
	100.00		Blank + IL-1α	LPC + IL-1α	HPC + IL-1α
	0.00	75.00	Blank + RPH-104	LPC + RPH-104	HPC + RPH-104
	100.00		Blank + IL-1α + RPH-104	LPC + IL-1α + RPH-104	HPC + IL-1α + RPH-104
IL1-β	0.00	0.00	Blank	LPC	HPC
	100.00		Blank + IL-1β	LPC + IL-1β	HPC + IL-1β
	0.00	75.00	Blank + RPH-104	LPC + RPH-104	HPC + RPH-104
	100.00		Blank + IL-1β + RPH-104	LPC + IL-1β + RPH-104	HPC + IL-1β + RPH-104
IL1-Ra	0.00	0.00	Blank	LPC	HPC
	500.00		Blank + IL-1Ra	LPC + IL-1Ra	HPC + IL-1Ra
	0.00	75.00	Blank + RPH-104	LPC + RPH-104	HPC + RPH-104
	500.00		Blank + IL-1Ra + RPH-104	LPC + IL-1Ra + RPH-104	HPC + IL-1Ra + RPH-104

**Table 3:** Mean OD values obtained during method sensitivity evaluation.

Sample		Mean OD	CV, %	Sample	Mean OD	CV, %
Analyst 1				Analyst 2		
Dilution series 1	Std.1	3.108	0.23	Std.1	2.332	16.50
	Std.2	2.264	1.19	Std.2	1.682	2.94
	Std.3	1.097	6.13	Std.3	0.877	0.73
	Std.4	0.609	7.79	Std.4	0.649	7.41
	Std.5	0.260	19.04	Std.5	0.268	16.36
	Std.6	0.145	0.98	Std.6	0.167	6.37
	Std.7	0.075	0.00	Std.7	0.049	8.66
	Std.8	0.055	3.89	Std.8	0.039	10.88
	Std.9	0.050	2.83	Std.9	0.043	4.99
	Std.10	0.037	3.82	Std.10	0.030	7.19
Dilution series 2	Std.1	3.131	0.09	Std.1	2.322	3.93
	Std.2	1.947	11.11	Std.2	1.648	2.57
	Std.3	0.956	8.81	Std.3	0.779	1.82
	Std.4	0.573	17.52	Std.4	0.554	2.81
	Std.5	0.227	6.56	Std.5	0.222	2.87
	Std.6	0.130	8.70	Std.6	0.164	8.62
	Std.7	0.064	19.89	Std.7	0.053	1.35
	Std.8	0.043	9.87	Std.8	0.043	3.29
	Std.9	0.043	11.65	Std.9	0.042	16.84
	Std.10	0.031	2.32	Std.10	0.033	15.23
Dilution series 3	Std.1	2.574	1.68	Std.1	2.068	2.87
	Std.2	1.889	1.98	Std.2	1.491	12.90
	Std.3	0.965	3.96	Std.3	0.858	4.78
	Std.4	0.451	4.39	Std.4	0.561	7.44
	Std.5	0.267	16.19	Std.5	0.226	18.15
	Std.6	0.154	18.89	Std.6	0.144	8.38
	Std.7	0.045	11.12	Std.7	0.047	7.60
	Std.8	0.037	11.47	Std.8	0.038	13.20
	Std.9	0.035	4.04	Std.9	0.024	5.89
	Std.10	0.020	3.63	Std.10	0.021	6.73

**Table 4:** Method sensitivity and LPC concentration calculations.

Dilution Series No.	Standard with a Mean OD Value Higher than PSCP, ng/mL	Standard with a Mean OD Value Higher than PSCP (Log Form)
1	50	1.699
2	75	1.875
3	75	1.875
4	75	1.875
5	75	1.875
6	100	2.000
Mean (log)		1.867
S.D. (log)		0.096
Sensitivity, ng/mL		114.9
LPC, ng/mL		154.9

**Table 5:** S/N ratio calculated for all standards with various anti-RPH-104 antibody concentrations.

Anti-RPH-104 Antibody Concentration, ng/mL	S/N Ratio					
	Series 1	Series 2	Series 3	Series 4	Series 5	Series 6
(Std_1)	89.439	90.101	74.058	68.254	67.946	60.527
(Std_2)	65.151	56.029	54.345	49.229	48.234	43.639
(Std_3)	31.554	27.496	27.770	25.654	22.800	25.112
(Std_4)	17.511	16.489	12.978	18.995	16.215	16.405
(Std_5)	7.482	6.518	7.669	7.844	6.483	6.615
(Std_6)	4.173	3.741	4.417	4.873	4.800	4.200
(Std_7)	2.158	1.842	1.281	1.434	1.537	1.361
(Std_8)	1.568	1.237	1.065	1.141	1.259	1.098
(Std_9)	1.439	1.223	1.007	1.244	1.229	0.702
(Std_10)	1.065	0.878	0.561	0.863	0.951	0.615

**Table 6:** Analytical method selectivity evaluation.

	Sample		Mean OD	CV, %	Mean OD	CV, %	% Inhibition
	**Screening Assay**				**Confirmatory Assay**		
Plate 1	Set 1	HPC_1	0.458	2.63	0.029	2.48	93.770
		LPC_1	0.052	6.87	0.023	6.15	55.340
		NQC1	0.027	15.71	0.024	3.01	12.963
		NQC2	0.024	3.01	0.022	9.87	8.511
	Set 2	HPC_2	0.307	11.30	0.023	0.00	92.496
		LPC_2	0.042	5.11	0.024	15.04	43.373
		NQC3	0.027	2.67	0.022	6.43	16.981
		NQC4	0.028	2.57	0.023	9.43	18.182
	1	NC1	0.026	5.44	0.024	11.79	7.692
		HPC1	0.388	1.28	0.028	7.71	92.903
		LPC1	0.049	10.21	0.024	0.00	50.515
	2	NC2	0.024	3.01	0.023	9.43	4.255
		HPC2	0.317	7.82	0.021	10.35	93.523
		LPC2	0.039	5.51	0.023	3.14	41.558
	3	NC3	0.020	3.63	0.017	8.32	12.821
		HPC3	0.346	10.63	0.022	6.43	93.642
		LPC3	0.037	5.81	0.022	16.44	41.096
	4	NC4	0.039	9.18	0.025	11.31	35.065
		HPC4	0.400	13.44	0.025	5.66	93.750
		LPC4	0.044	0.00	0.023	15.71	48.864
	5	NC5	0.027	2.67	0.024	9.03	11.321
		HPC5	0.307	2.54	0.023	6.15	92.496
		LPC5	0.042	3.37	0.023	12.30	45.238
Plate 2	6	NC6	0.036	3.93	0.030	2.40	18.056
		HPC6	0.607	6.18	0.034	8.32	94.394
		LPC6	0.069	9.29	0.035	2.05	49.635
	7	NC7	0.037	5.81	0.031	2.32	16.438
		HPC7	0.468	1.66	0.028	12.86	94.118
		LPC7	0.058	12.19	0.025	8.66	57.759
	8	NC8	0.030	4.71	0.024	3.01	21.667
		HPC8	0.464	5.79	0.031	2.32	93.427
		LPC8	0.067	10.55	0.026	8.32	61.940
	9	NC9	0.034	6.33	0.030	7.19	11.940
		HPC9	0.519	13.35	0.034	14.78	93.545
		LPC9	0.061	1.17	0.026	8.32	57.851
	10	NC10	0.028	10.10	0.023	6.15	17.857
		HPC10	0.383	10.17	0.026	5.44	93.203
		LPC10	0.053	10.67	0.023	9.43	57.547
	Set 1	HPC_1	0.597	6.99	0.034	8.32	94.300
		LPC_1	0.066	5.40	0.031	4.56	52.672
		NQC1	0.034	6.33	0.031	0.00	7.463
		NQC2	0.033	6.53	0.029	0.00	10.769
	Set 2	HPC_2	0.416	14.28	0.031	0.00	92.548
		LPC_2	0.055	9.08	0.029	12.41	47.706
		NQC3	0.034	16.64	0.028	0.00	17.647
		NQC4	0.034	4.16	0.033	2.18	4.412

**Table 7:** Calculations and results of method drug tolerance evaluation to the presence of RPH-104.

Sample		Mean OD	CV, %
Set 1	HPC_1	0.563	0.75
	LPC_1	0.069	10.25
	NC1	0.039	10.88
	NC2	0.037	7.64
Set 2	HPC_2	0.470	0.60
	LPC_2	0.066	5.40
	NC3	0.051	13.86
	NC4	0.036	1.99
1		0.129	2.19
2		0.050	11.31
3		0.050	4.29
4		0.039	12.86
5		0.039	12.86
6		0.148	2.87
7		0.048	4.47
8		0.043	6.58
9		0.041	3.45
10		0.041	6.90
11		0.200	6.36
12		0.070	17.30
13		0.057	4.96
14		0.048	7.44
15		0.046	9.22
16		0.424	3.84
17		0.096	4.42
18		0.086	11.51
19		0.072	3.93
20		0.075	9.43
21		0.908	0.08
22		0.166	8.12
23		0.120	6.51
24		0.094	6.02
25		0.088	4.82

**Table 8:** Results of the method specificity evaluation.

Sample	Anti-RPH-104 Antibody Concentration, ng/mL	RPH-104 Concentration, µg/mL	Mean OD	S.D.	CV, %
HPC-set 1	2500.00	0.00	0.907	0.025	2.7
LPC-set 1	155.00	0.00	0.086	0.008	9.1
NC1-set 1	0.00	0.00	0.044	0.001	3.2
NC2-set 1	0.00	0.00	0.048	0.006	11.8
Blank	0.00	0.00	0.048	0.003	5.9
Blank + IL-1α	0.00	0.00	0.048	0.001	2.9
Blank+ RPH-104	0.00	75.00	0.044	0.006	12.9
Blank + IL-1α + RPH-104	0.00	75.00	0.044	0.002	4.9
LPC	155.00	0.00	0.115	0.001	0.6
LPC + IL-1α	155.00	0.00	0.097	0.012	12.5
LPC + RPH-104	155.00	75.00	0.057	0.001	2.5
LPC + IL-1α + RPH-104	155.00	75.00	0.056	0.004	6.4
HPC	2500.00	0.00	0.916	0.016	1.7
HPC + IL-1α	2500.00	0.00	0.802	0.003	0.4
HPC + RPH-104	2500.00	75.00	0.176	0.003	1.6
HPC + IL-1α + RPH-104	2500.00	75.00	0.117	0.002	1.8
Blank	0.00	0.00	0.048	0.001	2.9
Blank + IL-1β	0.00	0.00	0.049	0.003	5.8
Blank + RPH-104	0.00	75.00	0.047	0.001	1.5
Blank + IL-1β + RPH-104	0.00	75.00	0.049	0.003	5.8
LPC	155.00	0.00	0.106	0.000	0.0
LPC + IL-1β	155.00	0.00	0.106	0.008	8.0
LPC + RPH-104	155.00	75.00	0.059	0.001	1.2
LPC + IL-1β + RPH-104	155.00	75.00	0.053	0.000	0.0
HPC	2500.00	0.00	0.851	0.004	0.4
HPC + IL-1β	2500.00	0.00	0.878	0.045	5.1
HPC + RPH-104	2500.00	75.00	0.165	0.014	8.6
HPC + IL-1β + RPH-104	2500.00	75.00	0.170	0.028	16.3
Blank	0.00	0.00	0.050	0.001	1.4
Blank + IL-1Ra	0.00	0.00	0.044	0.006	14.6
Blank + RPH-104	0.00	75.00	0.049	0.000	0.0
Blank + IL-1Ra + RPH-104	0.00	75.00	0.045	0.002	4.8
LPC	155.00	0.00	0.067	0.001	1.1
LPC + IL-1Ra	155.00	0.00	0.101	0.002	2.1
LPC + RPH-104	155.00	75.00	0.059	0.005	8.5
LPC + IL-1Ra + RPH-104	155.00	75.00	0.062	0.003	4.6
HPC	2500.00	0.00	0.956	0.008	0.9
HPC + IL-1Ra	2500.00	0.00	0.935	0.034	3.6
HPC + RPH-104	2500.00	75.00	0.185	0.008	4.6
HPC + IL-1Ra + RPH-104	2500.00	75.00	0.170	0.003	1.7
HPC-set 2	2500.00	0.00	0.404	0.039	9.6
LPC-set 2	155.00	0.00	0.069	0.002	3.1
NC1-set 2	0.00	0.00	0.042	0.004	10.1
NC2-set 2	0.00	0.00	0.041	0.000	0.0

**Table 9:** Intra-day precision results.

Sample (Screening)	Mead OD	S.D.	CV, %
HPC	0.454	0.059	13.05
LPC	0.054	0.004	7.44
Sample (Confirmatory assay)	Mean % Inhibition	S.D.	CV, %
HPC	92.074	0.757	0.82
LPC	46.103	2.133	4.63

**Table 10:** Inter-day precision results.

Sample (Screening)	Mead OD	S.D.	CV, %
HPC	0.417	0.036	8.74
LPC	0.052	0.005	9.42
Sample (Confirmatory assay)	Mean % Inhibition	S.D.	CV, %
HPC	93.000	1.124	1.21
LPC	50.798	5.608	11.04

**Table 11:** CV values calculated for bench-top (BTS) and freeze-thaw (F/T) sample stability assessment.

Sample	Mean OD	S.D.	CV, %
HPC_BTS15	0.476	0.011	2.27
LPC_BTS15	0.054	0.006	10.61
HPC_F/T	0.393	0.016	4.02
LPC_F/T	0.049	0.005	10.46

**Table 12:** CV values calculated for 30-, 60-, 209-, and 414-day long-term stability (LTS) evaluation.

Time Point	Sample	Mean OD	S.D.	CV, %
30 days	HPC_LTS	0.835	0.035	4.15
	LPC_LTS	0.092	0.002	2.46
60 days	HPC_LTS	0.722	0.044	6.07
	LPC_LTS	0.081	0.003	3.12
209 days	HPC_LTS	1.144	0.072	6.29
	LPC_LTS	0.119	0.003	2.89
414 days	HPC_LTS	0.787	0.122	15.50
	LPC_LTS	0.074	0.013	17.60

**Table 13:** Brief summary of sample analysis.

Assay Format	Samples Analyzed (N)	Results
Screening assay	201	10 screening positives
Confirmatory assay	10	6 confirmed positives
Titer assay	6	Titers for samples: 1:30, 1:30, 1:30, 1:60, 1:60, 1:960

## Data Availability

Not applicable.
